# Phage Therapy: Application and Related Problems—A Review

**DOI:** 10.3390/life16010057

**Published:** 2025-12-30

**Authors:** Katharina Sippel, Branko Velimirov

**Affiliations:** Section of Microbiology and Molecular Biology, Medical Faculty, Sigmund Freud University, Freud Platz 3, 1020 Wien, Austria; sippel.katharina@web.de

**Keywords:** bacteriophage, clinical trials, case reports, antibacterial agents, innate and adaptive immunity, phage cocktails, tolerability, safety

## Abstract

Bacteriophages, viruses that target bacteria, offer a promising alternative to antibiotics in the face of escalating bacterial resistance. Despite their discovery over a century ago, their widespread adoption has been impeded by regulatory challenges, limited funding, and the dominance of antibiotics. This review evaluates the current status of phage therapy by examining a comprehensive literature search, applying predefined inclusion and exclusion criteria. The review assesses selected scientific reports and clinical studies for their safety and efficacy profiles. Our findings indicate that advancements in phage therapy involve critical steps such as rapid bacterial detection, effective isolation, production, purification of phage preparations, and understanding their interactions with the host. Clinical studies generally show promising safety profiles with fewer adverse events compared to controls, and some trials suggest efficacy even at lower phage titers. Case reports further highlight phage therapy’s potential, demonstrating high success rates and minimal adverse events, although caution is advised due to potential biases. Despite promising results, significant research gaps remain, primarily due to the limited number of large-scale, well-designed clinical trials.

## 1. Introduction

In times of rising multi-drug resistance (MDR) in bacteria, bacteriophages are being proposed as a serious alternative to antibiotics [1]. Bacteriophages are viruses that are the natural predators of bacteria [2]. Their discovery is attributed to the independent work of Frederik Twort in 1915 and Felix d’Hérelle in 1917 [3]. Therapy with bacteriophages was not widely accepted in the West, and after the emergence of antibiotics in the 1940s, bacteriophage research was shifted to a more fundamental level [4]. Despite the increasing number of clinical trials, the understanding of bacteriophage interaction with mammalian tissue/cells is still largely unknown [5]. A deluge of seemingly contradicting reports stands in the way of any attempt to establish universal rules [1].

Also, as regulatory bodies have categorized bacteriophages as biological substances, they are covered by pharmaceutical laws [6], despite warnings from several scientists that this classification is improper [7]. Therapy with bacteriophages in Europe and the USA is currently only allowed under Article 37 of the Declaration of Helsinki, which permits experimental therapy in patients without other therapy options. However, this has limited clinical research evaluating phage therapy [8]. Only recently did the Food and Drug Administration (FDA) classify bacteriophages as “generally safe,” enabling more clinical trials with bacteriophages [8]. Though support for the practice has grown over the past 15 years, it has failed to advance [7], and there are still no dedicated guidelines for the manufacture of phage therapeutics [6]. Member states of the European Union are trying to find national solutions for phage therapy regulations [6,7,8], and such projects are already realized for Belgium, France, Georgia, and Russia. In Belgium, phage active pharmaceutical ingredients (APIs) to be used need a written quality judgement, followed by a test of an approved laboratory which may or may not issue a certificate to approve its use. In France, recommendations for using phage medicinal products were issued under the nominative Temporary Authorization for use (ATUn) by hospital pharmacies under the responsibility of a prescribing physician in the case of patients who are unable to participate in clinical trials [6]. In Georgia, ready-to-use phage medicines require marketing authorization, and customized phage preparation is achieved by authorized pharmacies, while phages for prophylactic and therapeutic use are included in the *Russian Pharmacopeia*. It has to be emphasized that to realize such regulations, phages must be produced according to good manufactory practice (GMP), ensuring product quality. For this purpose, the efficacy and safety of the phage must be demonstrated and documented via RCTs (Phases I to IV) [7].

Unfortunately, bacteriophage research has hardly been funded to date [9]. Strong patent protection and wide market distribution are not possible for customized medical goods derived from natural bacteriophages. Phage treatment does not, therefore, fit into the dominant pharmaceutical market paradigm [10]. This makes it more difficult for new start-ups to attract and keep investors, and it could also be the reason why big pharmaceutical companies are pulling out of the anti-infective market and are not making a commitment to phage treatment development [7]. Moreover, even if individual patients urgently need a specific therapy, the user base must typically be large enough to offset its development and marketing expenses [11].

This review, based on clinical trials, case reports, and systematic reviews from 2009 until May 2024 from several databases, aims to provide a comprehensive assessment of the current state of phage therapy in treating bacterial infections, highlighting both its potential and areas requiring further research, especially in terms of (1) safety (the prevention of harm to patients) and (2) efficacy (the ability of an intervention to produce the desired beneficial effect, often equated with effectiveness), while also elucidating the potential of bacteriophages and perspectives for the future.

In order to proceed systematically, we defined a number of criteria to decide on the integration of information from (a) clinical trials, (b) case reports, and (c) systematic reviews. Those criteria were the age of the patient, their gender, the route of administration, the outcome of the treatment, adverse events, and whether antibiotics were used or not.

## 2. Bacteriophages as Antibacterial Agents

Bacteriophages are limited to bacteria that possess a corresponding receptor [12]. The degree of specificity differs from bacteriophage to bacteriophage [12]. The role of the human phageome, even though present in all body niches, has so far only been sporadically investigated in microbiome studies. Nonetheless, there is published evidence that phages belonging to the *Podoviridae*, *Siphoviridae*, *Myoviridae*, *Microviridae,* and *Inoviridae* [13,14,15,16] have been detected in the human gut microbiome and mucosal surfaces, human blood, and feces [5,17]. It is worthwhile mentioning that *Inoviridae* carry ssDNA [17,18,19] and are not lytic, while all other groups are lytic dsDNA phages; the progeny virions of the Inoviridae are released from infected cells via extrusion without killing the host [18,19]. Major criteria for phage therapy encompass the selection of the phage for the specific bacterial infection. It is obvious that for the effectiveness of phage therapy, the identification of infecting bacteria is definitely a crucial initial measure.

### 2.1. Detection of Bacteria

Microbial culture media are still the “gold standard” for bacterial identification [19,20]. This technique typically involves isolating and cultivating the pathogen, which is then confirmed through biochemical and serological testing [21]. However, the drawback of this approach is that it can take up to seven days to obtain a response because certain bacteria take a while to display growth on culture media [22]. Therefore, many new strategies were and are being developed, as well as being tested to obtain more rapid confirmation of the pathogen (Table 1) [22]. Nonetheless, since all methods have their respective advantages and disadvantages, none is superior to the others, and rapid detection of bacteria remains a challenge.

### 2.2. Isolation and Production of Bacteriophages

The isolation of corresponding bacteriophages is the next step in guaranteeing the safety and efficacy of phage therapy [25]. Technically, samples can be taken from any surface or liquid (e.g., cell phones, toilet surfaces, trash receptacles, etc.) [26], but bacteriophages are usually best found in places where their hosts occur [27,28]. For phage therapy, the sample is often collected from sewage lines leading from a hospital or from patients themselves (i.e., human skin, fecal material, wound exudates, etc.) [27,29].

As therapy with bacteriophages is increasingly becoming an attractive new option in the fight against bacterial infections [30], phage production techniques that are both affordable and scalable are required to address the growing demand for faster bacteriophage supply to guarantee efficacy [31].

The three main steps in the production of bacteriophage therapeutics are amplification, concentration, and purification [32]. Otherwise, important factors for phage production are the populations of susceptible uninfected bacteria, phage-infected bacteria, and free bacteriophages [33]. Moreover, the phage burst size, the adsorption rate, and the multiplicity of infection (MOI), i.e., the correct ratio between bacteriophages and bacteria, are all critical elements in the phage manufacturing process [32]. Traditionally, bacteriophages are manufactured in stirred tank bioreactors or shake flasks [34].

Research on bacteriophage engineering is gaining a lot of attention and has the potential to significantly increase the antibacterial activity of bacteriophages used in phage therapy. Methods for engineering bacteriophages that have been effectively applied are presented in a number of studies and reviews [35]. This could enable their customization to go beyond bacterial resistance mechanisms and acquire desired characteristics that are not found in naturally occurring phage particles [32].

Nonetheless, rapid and effective production of bacteriophages on a large scale remains a challenge [34].

### 2.3. Purification of Bacteriophages

For a successful bacteriophage industry, the purification and concentration of bacteriophages are paramount, particularly to minimize safety concerns [36]. A significant obstacle is separating bacteriophages from bacterial debris like endotoxins, peptidoglycan, exotoxins, flagella, nucleic acids, and other compounds. If these impurities are not properly removed, they can cause severe inflammation and sepsis [37].

There are several methods to extract bacteriophages from bacterial cultures and environmental samples [38]. These include filtration, organic solvent clarification, polyethylene glycol (PEG) precipitation, and various forms of centrifugation, such as differential, density gradient, and caesium chloride (CsCl) gradient centrifugation [32,38].

However, it has been observed that purification procedures such as PEG or CsCl have a negative effect on some bacteriophages [38]. In addition, traditional techniques like ultracentrifugation often result in lower recoveries due to phage inactivation during pelleting [32]. To minimize these integrity losses, newer methods such as tangential flow filtration (TFF) and liquid chromatography have been developed [38].

Each purification strategy has drawbacks that can negatively affect bacteriophage samples, especially when multiple types of bacteriophages are present [38]. To ensure the safe use of bacteriophages, increasing the purity of bacteriophage therapeutics is critical as demand increases [39].

### 2.4. Bacterial Resistance

Bacteria naturally develop defence mechanisms against their enemy [40]. However, unlike antibiotics, bacteriophages can adapt and overcome these defences through spontaneous mutation and natural selection [41]. Still, bacterial resistance to bacteriophage infections limits the efficacy of phage therapies, especially when resistance mutations cause cross-resistance to multiple bacteriophages [42].

To overcome resistance, one approach is to introduce new bacteriophages that the bacteria are still susceptible to [40]. Another effective method is using phage cocktails, which consist of various types of bacteriophages [43]. Phage cocktails have been proven to impede resistance development by targeting multiple receptors, reducing the selection pressure on individual bacteriophages [42,44]. A different approach is to leverage the synergy between antibiotics and phage therapy, which can significantly enhance the clinical effectiveness of phage treatment. However, the exact mechanisms behind this synergy are often unknown and largely speculative [45].

In summary, while the understanding of bacterial defence mechanisms has advanced, there is still a lack of clarity about the ecological conditions that favour specific defences and their impact on bacterial and bacteriophage population dynamics [46]. Although bacterial defence systems have been investigated for a long time, there are still many knowledge gaps [47]. Therefore, studying the development of resistance, understanding their evolution, and knowing previous contact with other bacteriophages are important as they may determine bacteriophage efficiency [43].

### 2.5. Bacteriophages Eliciting Immune Responses in the Human Body

In the 20th century, bacteriophages were first isolated from human feces and have since been found throughout the body, including the gastrointestinal tract, blood, urine, skin, oral cavity, respiratory tract, and cerebrospinal fluid [5,48,49]. Bacteriophages are the most prevalent type of microbiota in the human body, thanks to their capability to pass through epithelial cells [48,50]. However, in contrast to the bacterial component of the human microbiome, the viral component has received less research attention [51]. Consequently, given their biology, bacteriophages necessitate investigation into their interactions with various body tissues and the immune system to ensure the safety and efficacy of phage therapy [52].

Because the innate immune response acts as the initial defence mechanism of the body against microorganisms, a therapeutic application of high-titer bacteriophages will certainly stimulate the host immune system [53]. While many specifics of these interactions remain unclear, multiple studies concur that the removal of bacteriophages by nonspecific defence and specific defence mechanisms may pose a problem in maintaining phage titers. It is established knowledge that processes such as phagocytosis, or clearance by Kupffer cells in the liver, as well as neutralization by anti-phage antibodies, pose a significant challenge in maintaining adequate phage levels for effective therapeutic outcomes [5].

Furthermore, although there is a theoretical risk of provoking severe immune responses like anaphylaxis with high phage concentrations, there have not been any reported cases of anaphylaxis due to phage therapy, and it does not seem to pose a significant safety concern [54].

Moreover, being constantly in contact with bacteriophages has led to the appearance of antibodies against bacteriophages in the blood [5]. This likely occurs due to regular exposure to bacteriophages and infections caused by the bacteria they target. This suggests that bacteriophages are well-tolerated by the immune system, or at least do not pose a safety risk. Rather, there is a risk that the attraction of macrophages by antibodies leads to the destruction of bacteriophages, thereby reducing the administered phage titer [5].

Also, bacteriophages have been found to directly affect immunity in ways that can be considered anti-inflammatory [49]. They can influence both innate and adaptive immunity, affecting processes like phagocytosis, cytokine responses, and antibody production. This modulation of the immune response can significantly influence the outcome of bacterial infections [49]. Phage cocktails were shown to modulate immune responses by inducing anti-inflammatory effects in their hosts, whereby the effects of phage antibiotic synergy as well as the phage’s genetic diversity [46,47,55] play a major role.

Not only did they counteract *P. aeruginosa* infections, but there was a reduction in the pro-inflammatory cytokines IL-1ß, IL-6, IL-8, as well as CXL12a [55]. An interesting finding was published by Cafora and co-authors [56] in the frame of a study on the effect of phages as anti-inflammatory agents on animal models. It was shown that the immunomodulatory effects on the immune system were elicited by phage proteins, while phage DNA had no effects on the animal models.

Bacteriophages may have their most immediate effect on the immune system during sepsis, where their ability to lyse bacteria can rapidly decrease the bacterial burden. Conversely, sepsis can also result from bacterial debris caused by bacteriophages lysing bacteria [51]. It is noted that the putative anti-inflammatory or immunosuppressive impact of bacteriophages is certainly not to be equated with the physiological effects of medications, recognized as being anti-inflammatory or immunosuppressive. Although the precise mechanisms by which bacteriophages might trigger anti-inflammatory reactions are still unknown, antimicrobial activity seems to be one of the contributing factors [49].

So far, bacteriophages appear to pose few safety concerns for the immune system, but maintaining sufficient phage titers remains a challenge [5]. Ultimately, the effectiveness of phage therapy depends primarily on the number of bacteriophages that can reach and destroy the target bacteria [57]. Consequently, it is clear that the administration routes for phages should be carefully selected in order to obtain a high level of effectiveness.

### 2.6. Routes of Administration

Clinical research has examined various methods of administering bacteriophages [52,57]. Topical application has shown success and safety in treating infected wounds, ulcers, or burns [57,58]. Oral administration is generally safe and comfortable for patients, but the acidic stomach environment can reduce bacteriophage efficacy [5]. Although active bacteriophages have already been detected in stools, it remains unclear how many of the phages are destroyed by the acidity of the stomach [57].

Administering phage preparations intravenously is an efficient way to treat widespread infections or cases of bacteraemia. It is generally considered both safe and effective [57]. Nonetheless, it should be taken into consideration that reduction in the phage concentration will take place via the reticuloendothelial system of the liver, harbouring mononuclear phagocytes, and by the spleen.

For respiratory epithelial tissues, research indicates that delivering enough bacteriophages is probably more effective through nasal or tracheal administration compared to other methods [5]. Inhaling bacteriophages holds potential and has demonstrated effectiveness in fighting multidrug-resistant bacterial lung infections in humans and mice [57]. However, a significant challenge lies in ensuring uniform placement of bacteriophages, as uneven distribution could reduce treatment efficacy [5].

The choice of a suitable route of administration for bacteriophages is crucial for the efficacy and tolerability of bacteriophages and ultimately determines the success of phage therapy. While various administration routes are safe and highly effective depending on the infection, oral administration generally appears to be the least efficient option for phage therapy [59].

## 3. Current State of Clinical Research

### 3.1. Randomized Controlled Trials (RCT) Testing Phage Therapy in Bacterial Infections

After filtering out ineligible data, the systematic search across three databases resulted in the inclusion of a total of ten records. All evaluated studies investigated the efficacy and safety of bacteriophages for the treatment of bacterial infections.

A therapeutic bacteriophage preparation known as “Biophage-PA” was investigated in the randomized controlled trial by Wright and co-authors [60] for treating chronic otitis media caused by antibiotic-resistant *Pseudomonas aeruginosa*. The control group consisted of patients receiving placebo material that was visually identical to the therapeutic bacteriophage preparations and could not be distinguished by clinicians. The RCT included a total of 24 participants. Both groups of patients received one dose of either the phage preparation or the placebo material, topically into the ear. The clinicians assessed safety and efficacy via adverse events, visual analogue scales of different parameters, and bacterial counts. The results of this study showed that the average bacterial count decreased faster and more efficiently in the group that received phage therapy. In the visual analogue scale (VAS), oral/aural temperatures, and diary cards, the phage-treated group demonstrated a significant clinical improvement from baseline, while the placebo group did not. However, at the end of the trial, a total of three out of twelve patients in both groups had complete eradication of the bacteria. Therefore, it has to be accepted that the odds of achieving a complete recovery with phage therapy are the same as compared to the control group. Adverse events were evenly distributed among both groups, occurring in about half of all patients.

In the prospective RCT by Rhoads and colleagues [61], topical application of the phage cocktail “WPP-201” was compared with the standard treatment (sterile saline) in patients with venous leg ulcers caused by several types of bacteria. The objective of the phase 1 trial was to test the safety of bacteriophage-based preparations for difficult-to-treat wounds. A total of 39 patients were included in this RCT. The control group (21 patients) was only treated with the standard sterile saline solution during debridement once a week over a period of 12 weeks. The intervention group (18 patients) received both the sterile saline solution and the phage cocktail. Safety and efficacy were assessed through adverse events, clinical status, blood tests, and photo documentation. No significant difference was determined for the frequency of adverse events, making both treatments equally effective. Interestingly, this trial used low titers of bacteriophages to assess safety as a primary concern and showed the efficacy of bacteriophages, even at lower concentrations. Nonetheless, it was concluded that a phase II efficacy study will be needed to evaluate the efficacy of the preparation.

The prospective RCT conducted by Sarker et al. [62] investigated the use of two different phage cocktails compared to a placebo in 120 male children aged 6–24 months with *E. coli*-induced diarrhoea. T4-like coliphages, a commercial Russian coliphage product, or a placebo was administered orally alongside the standard treatment for four days. Safety and efficacy were evaluated through lab results, clinical status, and fecal cultures. The results showed no significant difference between the three groups in any aspect. However, it should be noted that children without *E. coli* were included in the study, which could have distorted the results. Additionally, this study primarily focused on assessing the safety of bacteriophages. As a result, it utilized low phage titers and administered standard treatment concurrently, making the evaluation of efficacy rather difficult

The RCT by Jault et al. [63] investigated the use of a phage cocktail called “PP1131” for the treatment of burn wounds infected by *Pseudomonas aeruginosa*. The control group in this study received the standard of care (Sulfadiazine silver). A total of 25 patients were included in this trial. Although the intervention was initially concealed from both the patients and clinicians, differences in appearance made it possible for the clinicians to differentiate the treatments. However, the treatment allocation remained concealed for patients (due to anaesthesia) and for the researchers responsible for conducting the microbiological evaluations. Once daily, both groups received their treatment topically onto all infected wound sites. Seven days in total were spent administering all therapies, and observations were made for a total of 21 days. Safety and efficacy were assessed through adverse events, bacterial counts, and clinical examinations. The median time to a sustained semi-quantitative reduction in two or more quadrants of the daily bacterial load was noted by Jault and colleagues. They found that patients receiving bacteriophage treatment had a substantially longer median time of 143 h compared to those receiving standard care, who had a median time of 47 h. At the end of the treatment, a total of six out of twelve phage therapy patients and eleven out of thirteen standard of care patients had complete eradication of the bacteria and were considered as a successful outcome, making phage therapy less effective. However, the therapy with bacteriophages was considered safer, as twice as many adverse events were reported in the control group

Ooi et al. [64] conducted a prospective open-label trial on the use of the phage cocktail “AB-SA01” in patients with recalcitrant chronic rhinosinusitis caused by *Staphylococcus aureus*. The study included nine participants, evenly divided into three cohorts. All patients received two intranasal doses of the phage cocktail per day for seven days. To assess the safety and efficacy of the bacteriophages, researchers evaluated biochemistry tests, lab results, temperature measurements, physical examinations, and bacterial cultures. All patients showed a decrease in bacterial load and clinical improvement during and after phage therapy. By the end of the treatment, two patients had negative growth cultures. Six patients experienced mild adverse events, which were resolved the same day and were unlikely to be related to the bacteriophages.

The single-arm, non-comparative trial by Petrovic Fabijan and colleagues [65] tested the phage cocktail “AB-SA01” in 13 adults with *Staphylococcus aureus*-induced bacteremia. All patients received bacteriophages twice daily for 14 days in combination with antibiotics. Clinicians assessed bacterial load, lab results, and clinical examinations. The results showed a decrease in bacterial load and a decline in inflammatory markers in all participants after phage therapy. Moreover, no adverse events were reported. Nevertheless, two patients withdrew from care; one patient died after 28 days, and another died after 90 days. Both deaths were unrelated to the phage therapy. In the end, seven patients fully recovered.

Leitner et al.’s [66] RCT assessed the efficacy of intravesical phage therapy in treating urinary tract infections brought on by various bacteria. In this trial, there was a total of two control groups for comparison: one receiving bladder irrigation (placebo) and the other group receiving antibiotics (ceftriaxone, amoxicillin + clavulanic acid, or ciprofloxacin). The study included a total of 97 participants. Due to the different route of administration, the intervention of the antibiotics control group could not be masked. As the phage cocktail “Pyophage” and the placebo were made in identical vials and supplied in the same manner, it was possible to blindly administer both to every participant. The antibiotics group started receiving antibiotics 60 min before transurethral resection of the prostate (TURP), while the phage and placebo group received intravesical administration of either Pyophage or the placebo twice daily via suprapubic catheter for a total of seven days. No intravesical therapy was performed in the antibiotics group. Clinicians assessed safety and efficacy through adverse events, bacterial load, and clinical examinations. It is important to note that bladder irrigation may not be considered a placebo as labelled, as studies have demonstrated that daily bladder irrigation with tap water can decrease the bacterial burden [66]. Ultimately, both phage therapy and bladder irrigation showed about the same level of effectiveness. However, the group treated with antibiotics showed the greatest improvement, with a slightly higher number of successful treatments. Overall, phage therapy did not show superiority in the treatment of bacterial infections. Despite this, it is noteworthy that phage therapy had again only half the number of adverse events compared to each group, underscoring its safety.

The RCT by Dobretsov et al. [67] investigated the use of phage therapy in 40 patients suffering from chronic rhinosinusitis with nasal polyps. One group of patients received “Otofag” administered intranasally, a cocktail containing 32 types of bacteriophages, while the other group received a placebo. Both treatments were given twice a day for a total of 10 weeks. Bacteriological examinations and immunological analyses were performed to evaluate safety and efficacy. In summary, while the control group experienced very little to no improvement, the phage therapy patients achieved complete eradication of *Streptococci* after ten days, with a significant reduction in *Enterobacteria*, but no change in *Staphylococci*. Immunologically, there was no difference between the two groups. Adverse events were not mentioned. The authors themselves noted that their study lacks clinical and morphological assessment, which is a potential source of bias.

In their non-randomized prospective open-label trial with a historical control group (a group of patients from a past study), Fedorov and colleagues [68] evaluated the efficacy and safety of local phage therapy in 45 adults diagnosed with deep periprosthetic hip joint infection caused by *Staphylococcus aureus*. All patients received systemic antibiotics. During the implantation of cemented endoprosthesis, the intervention group underwent local commercial staphylococcal bacteriophage therapy, while the control group received local antibiotic administration. Safety and efficacy were evaluated through laboratory analyses, monitoring of adverse events, microbiological assessments, and cultures. By the study’s conclusion, all patients had fully recovered, except for one patient who was excluded due to the absence of *Staphylococcus aureus*. However, during the one-year follow-up period, relapse was found to be eight times more likely in the control group compared to the phage therapy group, indicating the superior long-term effectiveness of phage therapy. Additionally, only mild adverse events were reported in two recipients of phage therapy, characterized by a transient increase in temperature following phage administration, underscoring the safety profile of bacteriophages. It is important to acknowledge potential factors that may have influenced the study results. The historical nature of the control group precluded blinding and randomization, which could introduce bias into the findings.

Samaee and colleagues [69] performed an RCT on the use of a phage cocktail for treating bacterial pneumonia. The study included 60 patients who had to have concomitant moderate-to-severe COVID-19 and test positive for one or more of the following bacteria: *Pseudomonas aeruginosa*, *Acinetobacter baumannii*, or methicillin-resistant *Staphylococcus aureus* (ATCC No 33591). A phage cocktail or an identical-looking placebo was administered via inhalation every 12 h for 7 days. The authors assessed respiratory rate per minute, O2 saturation, CT scans, lab results, clinical examinations, adverse events, and bacterial load. The evaluation showed that the phage therapy group required significantly less hospitalization and intubation and had significantly more negative cultures at the end of the treatment, demonstrating superior efficacy compared to the control group. Patients who received the placebo experienced more symptoms, and only half of them showed negative bacterial cultures in the end. No adverse events were reported. However, the inclusion of patients who were lost to follow-up or had discontinued the study limits the validity of the results.

A notable observation is that many of the studies focused on phase 1 safety assessments, where the titer might not have been optimized for therapeutic efficacy. However, despite this, the results consistently showed a positive impact of bacteriophages across nearly all of the studies. Remarkably, in some instances, the phage-treated group exhibited superior outcomes compared to the control groups.

It is striking that there is not only a scarcity of studies on phage therapy but also a notable lack of participants in the majority of these studies. With such limited data, drawing definitive conclusions becomes challenging, if not questionable in terms of the reliability of the evidence. Nevertheless, adverse events were seldom reported across these studies, and those that were documented were predominantly categorized as mild and swiftly resolved. Yet, the extent to which bacteriophages directly contributed to these events remains uncertain.

Overall, bacteriophages have consistently demonstrated safety across all studies. While their effectiveness varies (Table 2), some studies show that bacteriophages can be efficient against bacterial infections.

### 3.2. Case Reports (CR) Testing Phage Therapy in Bacterial Infections

After selectively excluding those studies which were not compatible with our criteria (see Introduction), a total of 79 patients from 58 case reports/series were included in this analysis [70,71,72,73,74,75,76,77,78,79,80,81,82,83,84,85,86,87,88,89,90,91,92,93,94,95,96,97,98,99,100,101,102,103,104,105,106,107,108,109,110,111,112,113,114,115,116,117,118,119,120,121,122,123,124,125,126,127,128]. Among these 79 patients, 49 were male (62.03%), and 30 were female (37.97%). The ages ranged from one year to 88 years, with a mean age of 51.77 years. All patients had infections caused by at least one bacterium and were treated with phage therapy. The most prevalent bacterium causing infections in this sample was *Staphylococcus aureus* (38.8/36.6%), followed by *Pseudomonas aeruginosa* (27.7/30%) and *Klebsiella pneumoniae* (11.1/13.3%) for males and females, respectively. All other pathogens remained below 10% (Table 3).

**Figure 1:**
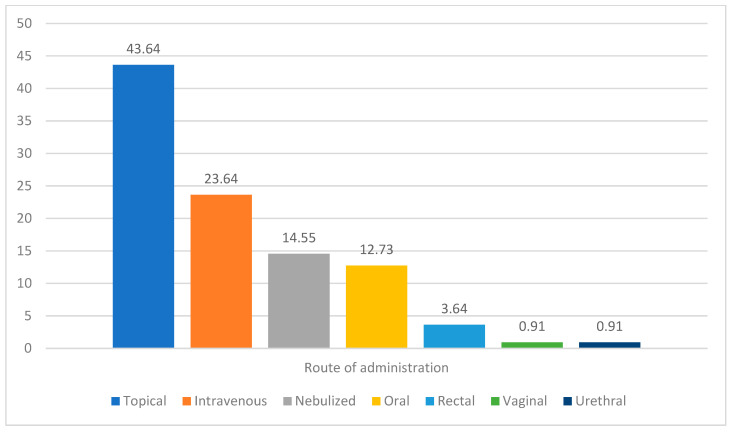
Percentage frequency of reported routes of administration in individual cases.

The route of administration varied among the patients, with many receiving bacteriophages through multiple routes (Figure 1). The most used form of application was topical administration, and 43.64% of the patients received bacteriophages topically. Intravenous administration was the second most common route, used by 23.64% of patients. These two routes were also the most frequently used in combination with other routes. Rectal, urethral, and vaginal routes were uncommon among the patients. This is likely because most patients did not have conditions suited to these forms of application.

Combining phage therapy with antibiotics shows promise in treating multi-drug resistant (MDR) infections. In the case studies reviewed, 62 out of 79 patients (78.48%) received both treatments, while 17 patients (21.52%) received only bacteriophages.

An important aspect regarding tolerability and safety is the occurrence of adverse events during phage therapy (Table 4). In total, 19 adverse events were reported among the patients. Of these, 16 events could possibly be related to phage therapy, while three were attributed to other causes, such as withdrawal of care despite clinical improvement or issues with the supply of bacteriophages.

The most common adverse events potentially related to phage therapy were elevated liver function tests and the development of resistance to bacteriophages, followed by fever. Other adverse events included increased pain, throat irritation, decompensation, pruritus, elevated CRP levels, and pneumothorax.

For these adverse events, bacteriophages (or potentially endotoxin levels) could not be definitively excluded as the cause, nor could they be conclusively identified as the reason, except in the case of resistance development. Nearly all patients experiencing these adverse events were receiving multiple treatments and were severely ill, which could also contribute to these events. In addition, all adverse events can be considered minor, as they were resolved quickly. Moreover, adverse events were more common in older patients. Particularly significant is the group of patients with elevated liver function tests, all of whom were also elderly (aged 64 years and above).

Notably, another interesting aspect concerns the outcomes of the patients (Table 5). Overall, 63 out of the 79 patients (63.29%) experienced complete clinical resolution after phage therapy. In 26 cases (32.91%), significant improvement was noted. Interestingly, the route of administration, the combination with antibiotics, or other variations in bacteriophage application had no discernible effect on the outcome; all patients had similar chances of success. Only in three patients (3.8%) did phage therapy have no effect, so their condition worsened over time. However, they were already critically ill before receiving phage therapy, and it is unlikely that phage therapy contributed to their deterioration.

Comparing the data of men and women in the case reports, it becomes evident that women experienced slightly more adverse events and slightly fewer clinical resolutions on average. Although the differences are minor, they should be interpreted with caution, as they may be attributed to the disparity in group sizes: significantly more men were included than women. This imbalance was not due to the exclusion of female cases but rather because male cases were considerably more frequent in the literature.

Interestingly, the same three pathogens appear to cause the most issues across all patients—*Staphylococcus aureus*, *Pseudomonas aeruginosa*, and *Klebsiella pneumoniae*—highlighting the potential threat they pose in the context of rising antimicrobial resistance

Still, the cases reviewed highlight several potential problems and biases. Selection and publication biases likely overestimate the efficacy of phage therapy, as studies with positive outcomes are more often published. The lack of a control group, as well as concurrent treatments, limits the ability to definitively attribute patient improvements to phage therapy. Variability in administration routes and phage preparations further complicates outcome analysis, as these factors are not uniformly controlled. Moreover, while adverse events were generally minor, they cannot be conclusively linked to phage therapy due to the severity of patients’ conditions and multiple treatments.

In conclusion, phage therapy proved highly effective in treating MDR bacterial infections among the cases reviewed. Generally, patients tolerated phage therapy well, experiencing only short-lived and minor adverse events that could potentially be related to the treatment. Nevertheless, while the case series suggests promising results for phage therapy, these findings should be interpreted with caution due to potential biases.

### 3.3. Systematic Reviews of Phage Therapy in Bacterial Infections

As phage therapy has advanced over time, there has been a corresponding increase in clinical research exploring its potential against bacterial infections. Interestingly, the number of systematic reviews on this topic has long outpaced that of clinical trials. This ongoing discrepancy underscores the intense interest in phage therapy, the need for alternative therapy options in the treatment of bacterial infections, and the considerable challenges in conducting clinical research in this field. This overview synthesizes the findings from several recent systematic reviews, which explore various aspects of phage therapy, including its efficacy, safety, and the obstacles it faces in broader clinical application.

Gordillo Altamirano and Barr provide a comprehensive comparison of phage therapy and antibiotics, emphasizing the versatility of phage applications, from conventional therapy to bioengineered bacteriophages. They highlight the promise of safe and efficient phage therapy, especially in antibiotic-resistant cases, but also point out the inconsistencies in clinical trial outcomes. Their review underlines key challenges, such as regulatory barriers, the need for standardization, and the limited global access to phage therapies [129].

Kortright et al. [3] delve into the history and mechanisms of bacteriophages, particularly focusing on the lytic cycle and the specificity of phage–host interactions. They discuss the potential of phage therapy, especially when combined with antibiotics, but also acknowledge the variability in treatment outcomes and the challenges posed by bacterial resistance to phages. The review highlights both the promise and the limitations of phage therapy.

Steele, Stacey, de Soir, and Jones [130] concentrate on the safety and efficacy of phage therapy in treating bacterial skin infections. Their review supports the view that phage therapy is generally safe and effective, offering a cost-effective and easy-to-administer alternative to antibiotics. Melo et al. [131] review preclinical studies on phage therapy over the last decade, focusing on its potential against multidrug-resistant bacterial infections. They discuss the therapeutic potential and challenges, including the variability in phage efficacy and the complexities of bacterial resistance. They concluded that phage therapy is generally safe and efficient, but also addressed concerns like risks associated with immune responses and the release of bacterial toxins following phage-induced lysis of the bacteria.

Liu et al. [132] examine both the safety and toxicity of phage therapy, analyzing animal studies and clinical trials. They highlight the potential risks, particularly immune responses and toxicity, while acknowledging the general safety of phage therapy. Stacey and co-authors provide a detailed evaluation of the safety and efficacy of phage therapy, drawing on various clinical and safety trials. They note the variability in clinical outcomes and stress the importance of understanding the conditions under which phage therapy is most effective and safe [133].

Strathdee and colleagues review the historical context of phage therapy, its decline, and its recent revitalization. They highlight the challenges in developing phage therapy, including the need for extensive characterization of bacteriophages and the complexity of designing effective phage cocktails. The review emphasizes the potential of genetically modified or synthetic bacteriophages to overcome these challenges [134].

Zalewska-Piątek discusses the effectiveness and safety of phage therapy in cases where traditional antibiotics have failed, highlighting its specificity to bacterial hosts as a key advantage. However, the review also addresses significant challenges, including the need for customization for each infection, regulatory barriers, and the potential for phage resistance [135].

In summary, while phage therapy shows significant promise as a solution to antibiotic-resistant infections, the field faces numerous challenges, including inconsistencies in efficacy, safety concerns, and regulatory hurdles. The systematic reviews analyzed herein collectively call for more rigorous research, improved regulatory frameworks, and a deeper understanding of phage biology to fully realize the potential of phage therapy in clinical practice. Nonetheless, there is a consensus that bacteriophage therapy holds great promise in combating antibiotic-resistant bacteria.

## 4. Hurdles of Phage Therapy

As mentioned earlier, bacteriophages are currently classified as biological medicinal products, bringing them under pharmaceutical legislation [6] —despite objections from experts who consider this classification inappropriate [7]. While therapeutic phages must meet stringent quality standards similar to those for conventional drugs, specific regulatory guidelines for their production are still lacking [6].

The production of bacteriophages according to EU and U.S. regulations remains expensive and time-consuming [136]. Integrating phage therapy into established frameworks—such as clinical trial models, pharmacokinetics, and regulatory systems—continues to pose a major challenge [8].

Moreover, financial support is a critical barrier: research and development of new phages receive little funding, even though early-stage work (e.g., isolation and characterization) is essential before clinical use [9]. Natural phage preparations cannot be easily patented or broadly marketed, making them commercially unattractive within the pharmaceutical business model [10]. Ownership issues and lack of profit incentives also hinder investment and industry involvement, especially as large pharmaceutical companies increasingly exit the anti-infective market [7]. Additionally, economic viability typically requires a large enough patient base to justify development costs—even when an urgent individual need exists [11].

## 5. Discussion

We have already mentioned that rapid and accurate detection of bacterial pathogens is a fundamental prerequisite for the successful application of bacteriophages in therapy. This initial step ensures the efficacy of phage treatment but also plays a pivotal role in saving valuable time. Further research aimed at developing or refining faster diagnostic methods that outperform current standards and can be seamlessly integrated into clinical practice is imperative. Such advancements hold the potential to significantly enhance patient outcomes, not only by enabling the prompt administration of phage therapy but also by mitigating the risk of inappropriate antibiotic use and other therapeutic interventions.

Another consideration is the centralized production of phage preparations, typically confined to specialized laboratories equipped with the necessary facilities. Enhancing efficiency could involve decentralizing production to enable local manufacturing, expediting delivery to clinicians. Alternatively, the establishment of widely accessible phage banks could expedite supply, offering readily available bacteriophages. However, before widespread implementation of phage banks, a diverse range of phage preparations must be amassed. Safety concerns dictate the necessity of maintaining high phage titer while minimizing endotoxin levels from bacterial lysis. Achieving complete endotoxin eradication remains elusive; hence, regulations establishing permissible endotoxin levels compatible with effective phage titers are essential. Furthermore, novel or optimized purification methods are imperative for enhancing the efficiency of phage preparation purification.

A challenge encountered in phage therapy is the emergence of bacterial resistance to bacteriophages. Predicting the evolutionary dynamics of bacteriophages could offer valuable insights for optimizing safety and efficacy in clinical settings. Utilizing phage cocktails or combining bacteriophages with antibiotics are two strategies aimed at preventing resistance development. Phage cocktails appear more advantageous for treating minor-to-moderate bacterial infections, while combining bacteriophages with antibiotics may be preferable for severe or persistent infections. Further research is essential to determine the most effective approach for specific infections.

When examining bacteriophages’ interactions with human tissue, they appear to have no adverse effects. Given that bacteriophages naturally inhabit and interact with the human body, they generally pose minimal risk to human health. However, the release of cellular debris resulting from bacterial cell lysis could potentially present challenges. Additionally, the immune system’s ability to detect bacteriophages via Toll-like receptors (TLR), like TLR3 recognizing viral dsRNA, TLR7 and TLR8 detecting viral ssRNA, and TLR 9, which recognizes viral RNA and unmethylated CpG-DNA, destroys them via macrophages, thus hindering their efficacy [137,138,139,140]. Therefore, selecting the appropriate route of administration is crucial to ensure the rapid and effective delivery of bacteriophages to the site of infection.

To determine the optimal route of administration for each case, a comprehensive understanding of the interaction between bacteriophages and various human tissues is essential [52]. Nonetheless, the influence of anti-phage antibodies regarding the effectiveness of bacteriophage therapeutics remains unclear [141]. It is unclear whether humoral immunity exists against phage pools and the roles these antibodies have in modulating phage activity, phage transcytosis, or opsonization of pathogenic bacteria targeted by phages. Further research is warranted to explore the barriers bacteriophages may encounter within the human body, such as the blood–brain barrier, as well as to elucidate mechanisms for maintaining high phage titers and addressing potential immune responses. In this context, it is worth mentioning that a national phage bank has been established in Belgium and a foundation for innovative Phage Applications and Therapeutics (IPATH) was created at the University of California, San Diego [142]. As a consequence, phage therapy becomes a component of personalized medicine.

### 5.1. Clinical Trials

Considering the results of the clinical trials, they suggest that phage therapy can be considered mostly safe and at least partially effective. Only a few minor adverse events were reported across all studies, and these were rapidly resolved. Although these events occurred during phage therapy, the involvement of bacteriophages remains mostly unclear. Notably, compared to control groups, phage therapy generally demonstrated a positive safety profile, with negative events more frequently observed in control groups, including those receiving a placebo. This may suggest that bacteriophages reduce the probability of adverse events.

However, we wish to underline the fact that efficacy outcomes varied among the above-mentioned studies. At first glance, most studies indicate that phage therapy is effective in treating bacterial infections. Upon closer examination, several issues emerge. One problem is the presentation of data in a way that favours phage therapy. For instance, some authors reported only the mean reduction in bacterial load, which suggested the superiority of bacteriophages. However, a detailed analysis of individual patient data revealed no significant difference between groups. Another example is that in one trial, differences in time to recovery were reported as non-significant; however, a more detailed analysis of the data suggests that this conclusion may be questionable [63]. Now, when authors do not publish raw data and only mention their conclusions, it raises concerns about potential bias in favour of phage therapy.

Additionally, in some studies, patients received antibiotics simultaneously, complicating the evaluation of bacteriophage effectiveness. Nevertheless, it is noteworthy that in one study, the combination of phage therapy and antibiotics was superior to a combination of different antibiotics, suggesting a promising synergistic relationship in combating bacterial infections.

Surprisingly, there are instances where placebos were more effective than bacteriophages, which is illogical, as bacteriophages should at least have a placebo effect. Some trials showed bacteriophages to be equally effective as placebos, indicating no efficacy. On the other hand, there are studies that do demonstrate bacteriophage efficacy. In some cases, bacteriophages were as effective as standard treatments, superior to placebos, or showed significant clinical improvements in studies without control groups. However, it must be noted that many of these studies were phase 1 trials focusing on safety rather than efficacy, and they often employed low phage titers.

Overall, several issues arise from these studies. A significant concern is the overall small number of studies and the limited number of participants in most of them, increasing the likelihood that results are due to chance. Additionally, methodological problems, such as issues with blinding, randomization, and the inclusion of patients without compatible bacterial infections, further complicate the findings. Consequently, due to the potential biases in these studies, it is problematic to draw reliable conclusions regarding the safety and, particularly, the efficacy of bacteriophage therapy. Further research, especially randomized controlled trials with higher phage titers, larger sample sizes, and careful monitoring of phage clearance, is urgently needed.

### 5.2. Case Reports

The case reports indicate that bacteriophage therapy can be a safe and effective strategy for treating bacterial infections, including those caused by multidrug-resistant (MDR) strains. Adverse events designated as potentially phage-related have not been conclusively linked to bacteriophage therapy, but they cannot be entirely ruled out. Most of these events occurred in patients over 60 years of age, many of whom were receiving multiple treatments simultaneously. This suggests that these adverse events could be triggered by concurrent antibiotic treatment or may indicate that phage therapy is less safe in older patients. Future studies should investigate the safety of phage therapy across different age groups.

The most common potentially phage-related adverse events were increases in liver function tests and the development of bacterial resistance to bacteriophages. Notably, many patients with elevated liver function had prosthetic joint infections and received topical bacteriophage administration directly at the infection site during surgery. Further studies are needed to evaluate the relationship between increased liver function and phage therapy to ensure safety. The development of bacterial resistance to bacteriophages is a logical consequence of phage treatment. Interestingly, in one patient, bacteria lost resistance to antibiotics when they developed resistance to bacteriophages. This finding suggests a promising approach for combating MDR infections even in challenging cases.

The efficacy outcomes were remarkably positive. Only two patients showed no effect from bacteriophage therapy, while all other cases demonstrated either complete resolution of the illness or a significant positive impact. These outcomes are particularly noteworthy given that all patients were considered therapy-refractory cases due to article 37 of the Declaration of Helsinki, underscoring the potential of phage therapy in treating difficult-to-treat infections.

Case reports are subject to several biases, including selection bias, publication bias, and performance bias. The likelihood of publication bias is high, as very few negative outcomes related to phage therapy were reported among the 82 patients included. There may possibly be limited interest in publishing case studies of failed phage therapy. Performance bias is also a major concern, as patients were treated by different researchers without a standardized protocol. Additionally, the absence of a control group prevents a robust evaluation of safety and efficacy. However, until more rigorous studies are conducted, case analyses like this one may provide an initial understanding of the potential of bacteriophages in the context of rising MDR bacterial infections.

### 5.3. Systematic Reviews

Finally, a key insight from the systematic reviews on phage therapy is the broad consensus on its efficacy and safety, though not without some concerns. While phage therapy is generally regarded as safe and shows partial efficacy across the reviews, there is a clear and pressing need for more rigorous research, standardized clinical trials, and improved regulatory frameworks to fully realize its potential in clinical practice. Despite these challenges, the reviews strongly agree that bacteriophage therapy could play a crucial role in combating antibiotic-resistant bacteria, offering a viable alternative or complement to traditional antibiotic treatments.

## 6. Conclusions

Phage therapy represents a promising alternative for the treatment of bacterial infections, especially in the context of increasing antibiotic resistance. The body of research reviewed indicates that bacteriophages have the potential to be a safe and effective treatment modality. Despite the reported favourable outcomes of the therapy, underlying limitations of the approach need to be mentioned.

First, the development of rapid bacterial detection methods, the establishment of centralized or decentralized production facilities, regulatory reform, and financial support are critical steps toward mainstreaming phage therapy. Addressing safety concerns, particularly the management of endotoxin levels and the development of bacterial resistance, is crucial. Additionally, optimizing delivery methods and understanding the interaction of bacteriophages with human tissues, especially the immune system, can enhance therapeutic outcomes. Ideally, phage preparations should have the following properties: minimal impurities, production under stringent quality control protocols, resistance to the immune system, stability, safety, efficacy, good shelf life, and compatibility with other therapeutics.

Second, significant gaps in research remain, particularly regarding large-scale, high-quality clinical trials. The limited number of studies, small sample sizes, and methodological flaws such as issues with blinding, randomization, and patient selection undermine the reliability of the current evidence. Moreover, the simultaneous use of antibiotics in some studies complicates the assessment of phage therapy’s true efficacy. While some trials show promising results, others reveal inconsistencies and even contradictions, such as cases where placebos appear more effective than phage treatments.

Third, case reports suggest that phage therapy can be safe and effective, particularly for multidrug-resistant infections. However, these findings are prone to selection and publication biases and lack the rigor of randomized controlled trials. Consequently, while the initial results are encouraging, they should be interpreted with caution. Based on the frequency among the cases, studies on bacterial wound infections caused by multidrug-resistant *Staphylococcus aureus* hold the potential to include large sample sizes, providing more robust evidence through larger, well-designed trials.

Fourth, the systematic reviews highlight a general consensus on the potential of phage therapy as a safe and effective alternative or complement to antibiotics, while emphasizing the need for more thorough research and regulatory improvements.

In conclusion, despite their potential, we need well-designed clinical trials with larger sample sizes, higher phage titers, and robust methodologies to provide clearer insights into the therapeutic potential of bacteriophages.

## Figures and Tables

**Table 1:** Main advantages and disadvantages of the most promising methods for bacterial detection.

Method	Advantages	Disadvantages
Polymerase Chain Reaction (PCR)	➢ Faster, more accurate results [23] ➢ Potential for automatization [23] ➢ Reproducible and easy to use [23]	➢ Not able to differentiate viable from non-viable cells [21,23] ➢ Can only monitor one target [22]
Phage Amplification Assay (PA)	➢ High specificity [23] ➢ Low cost [23]	➢ Very variable sensitivity [23]
Matrix-assisted laser desorption ionization time-of-flight mass spectrometry (MALDI-TOF MS)	➢ Potential for automatization [20] ➢ Reproducible and easy to use [14]	➢ Can only identify highly abundant and purified bacterial samples [20] ➢ Lengthy procedure [20] ➢ Low positive rate of bacterial culture [20]
Enzyme-Linked Immunosorbent Assay (ELISA)	➢ Highly sensitive [22] ➢ Very specific detection [22]	➢ Large amount of time required to obtain highly specific results [22] ➢ Many steps and resources required [22].
Surface-enhanced Raman scattering (SERS)	➢ High speed [24] ➢ Simultaneous detection and characterization [24] ➢ Multiplex analysis [24] ➢ Comparatively low cost [24]	➢ Low sensitivity [24] ➢ Low susceptibility to matrix interference [24] ➢ Low accuracy [24]
Flow cytometry	➢ High sensitivity in short times [21]	➢ Time-consuming preparations which determine sensitivity [21] ➢ Cell subpopulations with similar marker expressions are difficult to differentiate [21] ➢ Can generate large volumes of data [21]
Optical biosensors	➢ Detects minimal refractive index changes [21] ➢ Offers quantitative and qualitative data [21] ➢ Simple and non-laborious sample preparation [21] ➢ Rapid detection time [21]	➢ Has a lack of sensitivity [21]
Bioluminescent sensors	➢ Could be used to detect bacterial ATP, which is an indicator of microbial contamination [21]	➢ Weak detection limits are given for Gram-negative bacteria [21]

**Table 2:** Overview of clinical trial information from 2009 to 2023.

Study Type	Pathogen/ Disease	Partici-pants	Intervention/ Control	Assessed Parameters	Outcome	Adverse Events	Problems/ Bias	Year/ Ref.
Randomized controlled double-blind trial	*Pseudomonas aeruginosa*/chronic Otitis	24 patients, ≥18 years old	Phage cocktail Biophage-PA/ Placebo	Adverse events, visual analog scales of different parameters, bacterial counts	Significant clinical improvements and decrease in bacterial load in the phage group but not superior in comparison with the placebo	No major adverse effects (only mild to moderate ones); same amount in both groups	Small sample size; reported results biased in favour of phage therapy	2009/ [60]
Prospective randomized controlled double-blind trial	*Pseudomonas aeruginosa*, *Staphylococcus aureus* and *Escherichia coli*/ Venous leg ulcers	39 patients, ≥18 years old	Phage cocktail WPP201/ 50 mL sterile saline (standard treatment)	Clinical status, adverse events, blood tests, photo documentation	No significant differences between either group	No difference between either group; none attributed to bacteriophages	Focus on safety and not efficacy; low titers; small sample size	2009/ [61]
Prospective randomized controlled trial	*Escherichia coli*/ Bacterial Diarrhea	120 patients, 6–24-month-old, male	Phage cocktail T4-like + standard treatment/ Phage cocktail Microgen Colipoteus + standard treatment/ Placebo + standard treatment	Clinical status, blood tests, fecal cultures, adverse events	No significant difference between the 3 groups	One adverse event in the T4 group and one adverse event in the placebo group, probably unrelated to treatment	Children with negative cultures were included; many did not have E. coli; no cultures from the upper intestine	2016/ [62]
Randomized controlled double-blind trial	*Pseudomonas aeruginosa*/ Burn wound infection	25 patients, ≥18 years old	Phage cocktail PP1131/ Sulfadiazine silver	Adverse events, bacterial counts, clinical examination, lab results	Probability of complete recovery twice as high in the control group as the phage group and average time to sustained semi-quantitative decrease in bacterial load was much longer (PT median 144 h, control group median 47 h)	Adverse events were less frequent in the bacteriophage group	Low phage titers; no blinding possible for clinicians; the phage group was on average older and sicker; small sample size; results biased in favour of phage therapy	2019/ [63]
Randomized controlled double-blind trial	*Staphylococcus aureus*/ Recalcitrant chronic rhinosinusitis	9 patients, ≥18 years old	Phage cocktail AB-SA01/no control	Biochemistry tests, blood tests, temperature, clinical status, bacterial cultures, adverse events	Decrease in bacterial load and clinical improvement in all patients	6 adverse events in 6 patients, mild and resolved the same day, unlikely due to bacteriophages	No control group; no statistical analysis; small sample size	2019/ [64]
Single-arm non-comparative trial	*Staphylococcus aureus*/ Bacteraemia	13 patients, at least 18 years old	Phage cocktail AB-SA01 during antibiotic treatment/no control	Clinical status, bacterial load, blood tests, adverse events	3 died during 28 days, 2 withdraw care, 8 survived 28 days, 1 died after 90 days, 7 survived day 90; decrease bacterial load and decline inflammatory markers after phage therapy	None	No control group; small sample size	2020/ [65]
Randomized controlled double-blind trial	*Enterococcus* spp., *Escherichia coli*, *Proteus mirabilis*, *Pseudomonas aeruginosa*, *Staphylococcus* spp., *Streptococcus* spp./Urinary tract infection	97 patients, ≥18 years old	Phage cocktail Pyophage/ Placebo/ Systemic antibiotics	Adverse events, bacterial load, clinical examination, lab results	Only half as many patients treated with bacteriophages experienced complete recovery compared to those in the placebo and antibiotic groups	Adverse events were twice as common in the control groups	Antibiotic treatment could not be blinded; no placebo; uneven distribution of patients	2021/ [66]
Randomized controlled double-blind trial	*Bacteroides* spp., *Escherichia coli* spp., *Haemophilus influenzae* spp., *Klebsiella* spp., etc./ Chronic rhinosinusitis	40 patients, ≥18 years old	Phage cocktail Otofag/ Placebo	Bacterial load, immunological analysis	In phage group Streptococcae eradicated after 10 days, number of enterobactae decreased, no change regarding Staphylococcae; no difference	Not mentioned	Lack of clinical and morphological assessment; small sample size	2021/ [67]
Non-randomized prospective open-label trial with historical control	*Staphylococcaceae*/ Periprosthetic hip joint infection	45 patients, ≥18 years old	Systemic antibiotics + local phage therapy (Staphylococcal bacterio-phage)/ Systemic antibiotics + local antibiotics	Clinical status, blood tests, microbiological tests, bacterial load	Phage therapy is more effective; the rate of relapses in the phage group was eight times less than that in the control group	Short increase in temperature in 2 patients	No blinding; no control; no randomi-zation	2023/ [68]
Randomized controlled double-blind trial	*Pseudomonas aeruginosa*, *A. baumanii*, *Staph. aureus*/ Bacterial pneumonia with moderate to severe COVID-19	60 patients, all ages	Phage Cocktail/ Identical Placebo	Vital signs, CT scan, blood tests, adverse events, bacterial load	Hospitalization, negative cultures, and duration of intubation in the phage group half as long as in the control group; successful treatment in the phage group, 26/30 patients, and in the control group, 15/30 patients	None	Patients who discontinued treatment were also evaluated	2023/ [69]

**Table 3:** Percentage frequency of bacterial pathogens in males and females derived from individual case reports.

Bacterial Pathogen	Males (N = 49)	Females (N = 30)
*Staphylococcus aureus*	38.89	36.67
*Pseudomonas aeruginosa*	27.78	30
*Klebsiella pneumoniae*	11.11	13.33
*Acinetobacter baumanii*	9.26	0
*Enterococcus faecium*	3.7	3.33
*E. coli*	3.7	3.33
*Achromobacter xylosidans*	1.85	3.33
*Proteus mirabilis*	1.85	0
*Staphylococcus mitis*	1.85	0
*Burkholderia dolosa*	0	3.33
*Achromobacter* spp.	0	3.33
*Burkholderia multivorans*	0	3.33

**Table 4:** Percentage frequency of adverse events; data derived from case reports.

Adverse Events	Males	Females
None	79.6	70
Unrelated to bacteriophages	4.1	3.33
Potentially related to bacteriophages	16.33	26.67

**Table 5:** Clinical outcome (%) of the case reports after administering phage therapy.

Clinical Outcome	Males	Females
Clinical resolution	65.31	60
Clinical improvement	30.61	36.67
No effect	4.08	3.33

## Data Availability

Not applicable.
